# Cyclodextrin Monomers and Polymers for Drug Activity Enhancement

**DOI:** 10.3390/polym13111684

**Published:** 2021-05-21

**Authors:** Adrián Matencio, Gjylije Hoti, Yousef Khazaei Monfared, Azam Rezayat, Alberto Rubin Pedrazzo, Fabrizio Caldera, Francesco Trotta

**Affiliations:** 1 Dip. Di Chimica, Università di Torino, Via P. Giuria 7, 10125 Torino, Italy; gjylije.hoti@unito.it (G.H.); yousef.khazaeimonfared@unito.it (Y.K.M.); azamrezayat1981@gmail.com (A.R.); alberto.rubinpedrazzo@unito.it (A.R.P.); fabrizio.caldera@unito.it (F.C.); 2 Department of Chemistry, Faculty of Science, Lorestan University, Khorramabad, Iran

**Keywords:** cyclodextrins, polymers, enhancers, stability, drug, bioactivity, review

## Abstract

Cyclodextrins (CDs) and cyclodextrin (CD)-based polymers are well-known complexing agents. One of their distinctive features is to increase the quantity of a drug in a solution or improve its delivery. However, in certain instances, the activity of the solutions is increased not only due to the increase of the drug dose but also due to the drug complexation. Based on numerous studies reviewed, the drug appeared more active in a complex form. This review aims to summarize the performance of CDs and CD-based polymers as activity enhancers. Accordingly, the review is divided into two parts, i.e., the effect of CDs as active drugs and as enhancers in antimicrobials, antivirals, cardiovascular diseases, cancer, neuroprotective agents, and antioxidants.

## 1. Introduction

Cyclodextrins (CDs) are well-known in the scientific community for their use to solubilize poorly soluble drugs [1]. Moreover, cyclodextrin (CD) presents intrinsic bioactivity to manage some diseases such as atherosclerosis or Niemann Pick type C [2,3]. Chemically, CDs are truncated, cone-shaped oligosaccharides made up of α-(1,4)-linked glucose units, obtained by the degradation of starch by the enzyme cyclodextrin glucosyltransferase (CGTAse). The most common CDs are the natural α, β, and γ-CD, which contain six, seven, and eight glucose units, respectively. The CD ring is a conical cylinder of an amphiphilic nature, with a hydrophilic outer layer (formed by the hydroxyl groups) and a lipophilic cavity [4,5]. When poorly soluble drugs are complexed with CD, it creates the so-called “inclusion complex” [6,7,8,9,10,11,12,13]. To improve the properties of CD monomers, different chemically obtained derivates (e.g., hydroxypropyl-β-CD or methyl-β-CD among others) and polymers have been shown to possess better capacities, such as complexation efficiency or release, than natural CDs [14,15,16].

**Figure 1:**
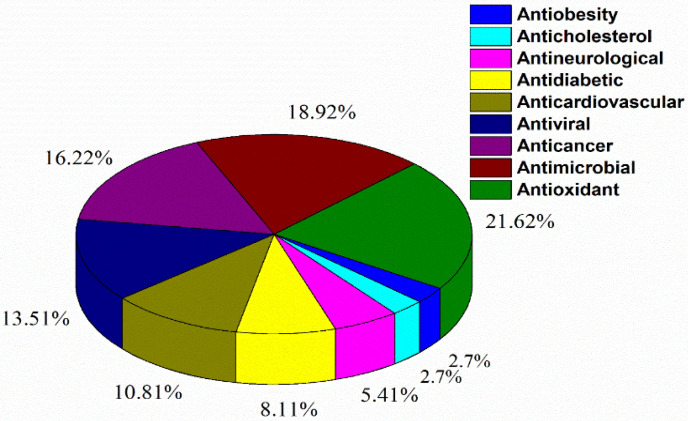
Distribution of biological applications of CD inclusion complexes reported in the literature (using keywords: “cyclodextrin inclusion complexes” in combination with “antimicrobial activity”, “cholesterol-related diseases”, “diet agent”, “antiviral activity”, “cardiovascular”, “neurological diseases”, “anticancer”, “antioxidant”, and “diabetes”). All publication data were obtained from PubMed, covering the period from 1978 until April 2021.

After the first applications of CDs in the pharmaceutical industry in the 1980s [17], a growing interest was observed for inclusion complexes with several applications. This was an introduction of a safe way to deliver drugs overcoming the downsides of traditional anticancer treatments as an example [18]. A recent search on PubMed, performed by us in May 2021, pointed to more than five thousand publications available since 1974 containing the keyword “cyclodextrin inclusion complexes”. The pie chart in Figure 1 presents the entries found by combining “cyclodextrin inclusion complexes” with “type of biological applications” (such as antimicrobial, anticancer activity, etc.). In this figure, the high percentages of all the publications dealing with antioxidant (21.62%), antimicrobial (18.92%), and anticancer activity (16.22%) show that these biological applications, among others, are the most extensively explored.

Moreover, an increase in the concentration of poorly soluble drugs increases their solubility and bioactivity [5,19]. Furthermore, the correct formulation might increase the effectiveness of therapies at the same concentration of the drug. In contrast to the effects of increasing the quantity of drug to achieve the dosage, some effects of complexation such as a higher bioaccessibility, drug stabilization, or target delivery may affect the whole effectivity of the therapy; CD monomers and polymers have demonstrated real capacities in this area [20,21]. Different reviews have been recently published about the capacities of CDs as drugs, in drug delivery, and their effects in membranes [1,2,22,23], the present review, therefore, presents a different point of view. It aims to provide deeply, a general overview of the use of CD monomers and polymers as enhancers for improving the bioactivities of the molecules at a fixed concentration, without undervaluing the effects of the concentration.

## 2. Cyclodextrin Monomers and Polymers as Drugs Themselves

In general, it is easy to find CD monomers as excipients in drug formulation. In some cases, CD itself is effective against the disease [2]. This section will try to describe some examples (Table 1) of this capacity.

### 2.1. Anticholesterol Effect

Perhaps the complexation of cholesterol is the principal application of CDs in pharma [24]; in this section, we take a look at different targets associated with this target and others:

For cholesterol-related diseases such as Niemann–Pick disease Type C (NPC, a rare disease resulting in the accumulation of lipids (generally cholesterol) in the cells [25]) or atherosclerosis (cholesterol accumulation in the wall of arteries), CDs are the most promising treatment for their capacity to complex the lipids and mobilize them [3]. It has been demonstrated that Mβ-CD and HPβ-CD reduce cholesterol accumulation [26,27,28,29]. Pilely et al., in 2019 discovered that α-CD and HPβ-CD can solubilize cholesterol crystals, reducing the complement-mediated inflammation by binding of C1q (via IgM) and ficolin-2 to cholesterol crystals, which resulted in reduced phagocytosis and reduced ROS production [30]. Moreover, different derivatives such as HPγ-CD (which cannot complex cholesterol) mobilize cholesterol; the treatment with HPγ-CD and HPβ-CD [31] induced the expression of protein-like LAMP-1, which is expressed in the lysosomal membrane. Cholesterol may be linked to this protein, thus facilitating its sequestration [29].

Different CD monomers and polymers are synthetized to optimize treatments: from 6-O-maltosyl-β-CD (G2-β-CD) [32], mono-lactose β-CD (Lac-β-CD), and multi-lactose (multi-lac-β-CD) [33] or octa-arginine derivative [34] CD monomers to CD-based polymers, formed by covalent bonds or CD-based polyrotaxanes (CDPRX) [35]. The principal advantage of the polyrotaxane polymer is that the cavity is covered by the polyrotaxane, improving its endocytosis, preventing the uptake of the cholesterol membrane, and reducing its toxicity [35]. Another interesting polymer is ORX-301, a pH-sensitive β-CD-based polymer with better pharmacokinetics and bioavailability [36].

On the other hand, the capacity to complex cholesterol was tested as a possible treatment against different agents. For example, they present an interesting antiviral effect against viruses with cholesterol in the membrane such as the influenza virus in vitro [37,38]. However, these results lack the foundations for producing novel therapies against the influenza virus. Based on this particularity, different materials designed to enhance their intrinsic activity, including a family of fullerene-cyclodextrin conjugates [39], pentacyclic triterpene-functionalized per-(2,3-di-O-methyl)-α-, β-, and γ-CD derivatives [40,41], or water-soluble β-cyclodextrin–glycyrrhetinic acid conjugates [42], were tested against the influenza virus. Similar results were observed in the case of herpes virus 1 [43], varicella-zoster virus (VZV) [44], and hepatitis C virus [45] or HIV infection [46], where novel, branched anti-HIV CDs were synthesized. The long-chain alkyl group penetrates and is fixed into the lipid bilayer of HIV and the sulfated maltoheptaose moiety electrostatically interacts with the HIV gp120 molecule [46,47]. Several of these viruses are also HS-dependent (generally heparan sulfate proteoglycans [48]). The novel, highly sulfonated CD derivatives (sodium undec-10-enesulfonate with different length chains) have been tested against the aforesaid viruses. The derivatives exhibited a broad-spectrum virucidal, irreversible mechanism of action, with high biocompatibility and acted as a barrier to viral resistance.

### 2.2. Cyclodextrins as an Active Diet Agent

Ingested CDs monomer can reduce hypercholesterolemia by reducing cholesterol absorption and even plasma cholesterol or triglyceride levels [49,50,51,52]. Concretely, α-CD was able to alter the gut microbiota, increasing the production of lactic acid and SCFAs. This had beneficial antiobesity effects by modulating the expression of genes related to lipid metabolism, indicating the prebiotic property of α-CD due to its metabolization [53]. The effect of γ-CD in the feed of mice was studied recently, the data indicated that dietary γ-CD leads to increased endurance—parameters such as traversed distance per night and general activity were increased; however, clear biochemical evidence was not found [54]. Finally, the EFSA permitted α-CD to be described as a dietary fiber, suitable for reducing postprandial glycemic responses due to its competitive inhibition of α-amylase [55].

## 3. Cyclodextrin Monomers and Polymers as an Enhancer of the Drug Effect

In this section, a summary of the different effects of CD monomers and polymer complexes is displayed (Table 1).

### 3.1. Antimicrobial Activity

In this field, several antimicrobial compounds present a hydrophobic nature, such as many essential oils with antimicrobial activity [56,57,58]. The encapsulation of those compounds in CD monomers and polymers can firstly enhance their aqueous solubility and maybe other properties. One example is the case of *trans*-anethone, whose properties were tested in the presence of CD monomers, showing not only better solubility but also superior UV-light and thermal stability [59]. In another study, an increase up to the limit of the solubility of the stilbene oxyresveratrol demonstrated increased antimicrobial capacity against *Escherichia coli* in solution [19].

The effect of CDs on the protection of different antimicrobials can increase the remaining bioactivity after different processes (UV, digestion, etc.). Further, a study introduced the capacity of CDs to maintain twice the concentration of the natural antimicrobial neochlorogenic acid [9]. In a further survey, β-CD and glucose were used as a stabilizer and reducing agent to synthesize silver particulars for bactericidal activity against the microorganism *E. coli* [60]. On the other hand, functional polystyrene (PS) fibers containing cyclodextrin–menthol inclusion complexes demonstrated an enhancement of thermal stability and durability [61]. In particular, the effect of the polymerization may improve the antimicrobial controlled release. For example, the natural antimicrobial carvatrol (essential oil of oregano) was complexed with β-CD polymers of polyethylene [57], chitosan [62], or cellulose [63], enhancing its antimicrobial activity. Indeed, the combinatorial use of chitosan or sodium alginate is quite effective to improve food packaging with CD/antimicrobial complexes [64,65]. Moreover, electrospun CD nanofibers also showed enhanced antimicrobial activities. In one study, the antifungal activity of HPβ-CD nanofibers with the drug thiram (an important protective fungicide in agricultural production) or thiabendazole was 1.32 and 1.83 folds of the untreated forms, respectively [66,67]. This system presented decent results not only for fungicides but also for antibacterial agents. The limonene complexes with HPβ-, Mβ-, or HPγ- as electrospun polymers presented better antibacterial activity against *E. coli* and *Staphylococcus aureus* [68].

Nevertheless, the material known as cyclodextrin-based nanosponges (CD-NSs, [15]) demonstrated interesting results to improve the controlled release of natural antimicrobials, for example with coriander essential, Babchi, or cinnamon oil [69,70,71]. The combination of CD-NSs and different antibiotic such as norfloxacin [72] showed an increase in the in vivo antibiotic capacity and permeability. The use of natural and simple derivatives as a carbon source might prevent the antibacterial effect. The use of citronellal with acarbose (added such as Glucobay^®^, an amylase inhibitor) was proposed as a solution [58]. Not only drugs but also antimicrobial enzymes can be complexed: In one study, functionalized CD-NSs were used to absorb lysozyme as an antimicrobial [73]. The possibility to modulate the charge or substituents in these materials is a good strategy to obtain an intrinsic antimicrobial activity; curiously, β-CD-based nanosponges crosslinked with carbonyldiimidazole presented antimicrobial activity [74]. Bearing the above in mind, this leads to increased utilization of these materials with antimicrobials.

### 3.2. Antiviral Activity

As mentioned above, CDs and CD-based polymers in their capacity to complex cholesterol can be used as antivirals. Moreover, the possibility to complex a drug increased the capacity of these materials to deliver a successful therapy. The capacity to increase the solubility of antivirals such as acyclovir, efavirenz, or lopinavir was tested using commercially available CD monomers (β-CD or HPβ-CD) with a 1:1 antiviral:CD ratio [75,76,77]. In particular, the antiviral ganciclovir, used against cytomegalovirus, demonstrated an in vitro antiviral potency complexed with β-CD in a 1:10 antiviral: CD ratio [78].

Additionally, several CD-based polymers have been tested in this field; for example, using CD-NSs of β-CD, the bioavailability of the HIV treatments efavirenz or rilpivirine was increased twice in comparison with free drug after oral administration to rats [79,80]. Further, the intrinsic low solubility of the drug nelfinavir, a HIV-protease inhibitor was increased using the same strategy [81]. The complexation of acyclovir, a well-known antiviral drug with CD-NSs was studied too; it was observed to have a slower release and enhanced antiviral activity against a clinical isolate of HSV-1 [82]. Other types of polymers, such as hyaluronic acid-CD covalent conjugates or HPβ-CD electrospun polymers, are developed to deliver acyclovir with good bioactivity/release and release, respectively [83,84]. Furthermore, sulfobutyl ether-β-CD decorated with a nanodroplet chitosan shell was employed to prepare an inclusion complex of acyclovir–cyclodextrin for the local treatment of HSV-2 infections. Antiviral activity was enhanced in the acyclovir-loaded nanodroplets compare to free drug against HSV-2 in cell cultures. This might be described as a higher intracellular accumulation of the drug in nanodroplet-treated cells than in free-acyclovir-treated cells [85]. Finally, the capacity to modulate the charge and the groups of CD-NSs was suggested as a possible COVID-19 treatment, alone or carrying some antivirals [86].

### 3.3. Cardiovascular Activity

Although the effect of CD monomers and polymers on cholesterol mobilization in atherosclerosis is detailed in Section 2, some drugs can be complexed with these molecules to be used as a therapy. Regarding hypertension, the complex of linalool with the monomer β-CD enhanced the decrease of arterial pressure in comparison with the free drug [87]. Similarly, the complexed monoterpene β-pinene also demonstrated a capacity to decrease the arterial pressure, a fact that it could not achieve as a free drug [88]. The increase in stability of these drugs, which are known as volatile compounds, justifies these promising results [58].

Hydrochlorothiazide (HTZ) is the only FDA-approved diuretic drug for children and is mainly consumed to treat hypertension, this drug presents low solubility and low stability in an aqueous solution [89]. Its complexation with β-CD monomers [90] in combination with solid lipid nanoparticles is used to solve these problems, and the results illustrated that the oral bioavailability of HTZ in both the diuretic effect and the sustained drug release was improved. Recently, the monomers HPβ-CD and SBEβ-CD were tested to prepare a combinatorial formulation with PVP polymers. The results showed that SBEβ-CD was more effective than HPβ-CD to solubilize and stabilize HTZ, and to increase its stability in the presence of PVP polymer [91].

A different strategy was proposed using α-CD monomers and polymers: the delivery of oxygen to limit hypoxia and reoxygenation injury [92,93]. Using the H9C2 cell line, three formulations—α-CD, branched α-CD polymer, and α-CD NS—were tested. Although the three formulations increased the recovery of the cell line, α-CD NS obtained the best result and showed a marked efficacy in controlled oxygenation, which suggests an interesting potential for future medical application.

### 3.4. Neurological Diseases

#### 3.4.1. Alzheimer’s Disease

Alzheimer’s disease (AD) results from an accumulation of β-amyloid peptides (AP) in the brain, which is linked to an abnormal cholesterol metabolism [94,95,96]. In this disease, CDs present engrossing possibilities; β-CD and HPβ-CD can bind AP directly to prevent aggregation and disaggregate [97,98,99]. Moreover, a co-assembly material between CD and calixarene is an anti-aggregation agent for AP tested in mice [100]. In other research, the conjugation of LVFFARK-NH2 (LK7) peptide to β-CD demonstrated a higher protective effect on AP-induced cytotoxicity and anti-aggregation capacity than LK7 alone [101].

Several molecules such as crocetin (CRT) and curcumin were complexed with γ- and HPβ-CD monomers to increase their delivery by intravenous injection or nasal administration [102,103] and to prevent oxidative damage in AD. In these particular trials, the delivery of curcumin complexed with HPβ-CD was compared with the chitosan-coated polylactic polymer complex. Both formulations displayed an anti-inflammatory effect at 20 μM CUR in BV-2 cells, which decreased TNF-α and IL-6 levels to approximately 70% and 40%, respectively. Moreover, although both materials increased the stability and capacities of curcumin, in vivo delivery of curcumin complexed with HPβ-CD displayed higher bioavailability than the polymer formulation [103].

#### 3.4.2. Parkinson’s Disease

Parkinson’s disease (PD) is caused by α-synuclein protein aggregation and misfolding [104]. It is reported that CD (in particular Mβ-CD) monomers present the capacity of complexing α-synuclein, preventing its aggregation [105]. The complexation of L-dopa, one of the most noteworthy treatments, is studied by several CD monomers and polymers [106,107]. The molecularly imprinted technique was used by Trotta and coworkers to create specific CD-NSs with a prolonged release profile than the nonimprinted NS. No degradation of the L-dopa hosted in NS was observed after long-term storage at room temperature [107].

### 3.5. Anticancer Activity

Several biological studies in vitro and in vivo were carried out to express the anticancer activity of complexes containing anticancer compounds [17]. The anticancer drugs, among plenty of others, such as camptothecin [108], curcumin [109], paclitaxel [110], tamoxifen [111], resveratrol [112,113], quercetin [114], temozolomide [115], doxorubicin [116], oxaliplatin [117], β-lapachone [118], N-biphenylnicotinamides (PTA34 and PTA73) [119], 13-cis-retinoic acid (13-cis-RA) [120], oxaliplatin [117], epothilone A [121], paclitaxel (PCX) [122], difluorinated curcumin (CDF) [123], niclosamide [124], are complexed with CDs and their derivatives to improve their efficacy, stability, solubility, and bioavailability; reduce their toxicity; and modify their physicochemical peculiarities [125], in comparison to their uncomplexed forms. After the identification of these new therapeutic anticancer strategies, of particular interest was also the noninclusion complex between CDs and riboflavin (RF) [126]. Riboflavin is well known for reducing the cancer risk in humans [127], but its application is limited because of relatively poor water solubility [126]. The complexation (CDs-RF) occurred because of the hydrogen bond formation between RF and the external rim of CDs. Several physicochemical approaches used in this study confirmed the formation of a noninclusion complex (CDs-RF) as an alternative mechanism to improve the biological activity of RF [126].

After the great interest presented by CD complexation, the dual approach of cyclodextrin and nanotechnology came as a novel plan for the more effective delivery of anticancer drugs [125]. Nanoparticles, liposomes, microspheres, hydrogels, and nanosponges were delivery systems with which the CDs were associated [18]. The evidence from the literature [128,129] highlights the modulation of the anticancer activity of 20 (S)-camptothecin (CPT) by hydrolysis of the ring E α-hydroxy δ-lactone moiety. Anticancer activity is related with the lactone, whereas the carboxylate is inactive and favored at physiological pH. As an important structural requirement for the successful interaction with the cancer cells, a closed lactone ring was considered. This is because of the inactivity of the ring-opened carboxylate at pH greater than 4 causing reduced potency in plasma. Therefore, significantly, a study developed the synthesis of water-soluble CD-based polymers containing pendant carboxylate groups that attach CPT on its 20-OH. This substitution resulted in the reduction of the lactone-ring opening, increasing in this way CPT’s anticancer activity [130]. CD-based polymers were further investigated as carriers for sorafenib, increasing its low solubility and reducing its toxicity [131]. Then, those polymers were also exploited to construct nanocarriers based on CD polymers endowed with an RGD peptide derivative for the targeted delivery of doxorubicin. It was observed that low-molecular-weight CD polymers may contribute to new tools for cancer therapy [132].

Since the crosslinking of CDs brings benefits to the CD-NSs [133], it was found to be a way to improve the performance of anticancer drugs. To advance our understanding of this development, the focus will be on camptothecin (CPT), an anticancer drug with severe toxicity [129], in spite of significant studies made on other drugs. As previously mentioned, attempts were continuously made to maximize therapeutic efficacy and minimize side effects of CPT starting from its inclusion complexes with CD [134] and its conjugation with CD polymers [130] to the use of CD nanosponge technology [135]. In vitro release studies are chosen as a point for comparison of the previously mentioned studies. Briefly, 38% *w*/*w* of CPT was loaded in a nanosponge and from in vitro release studies its slow release was observed without the initial burst effect. After 2 h, the percentage of CPT released was 4% and was significantly effective in reducing cell proliferation following 96 h treatment [135]. It demonstrated more prolonged release kinetics than with CPT conjugates (6–10 wt.% CPT loading), in which half of the total conjugated CPT was released after 32 and 59 h. This is related to the hydrolysis rate of CPT at pH = 7.4 [130], whereas CD–CPT inclusion complexes loaded 9% CPT and released 30% CPT after 2 h with burst effect [134].

These results represent progress toward the improvement of models for camptothecin delivery. Moreover, CD-NSs were also proposed as effective nanocarriers for the delivery of curcumin [136], paclitaxel [137], tamoxifen [138], resveratrol [139], oxyresveratrol [16], quercetin [140], doxorubicin [141], etc. In all these findings, an enhancement of the biocompatibility and aqueous solubility of those drugs was observed compared to CD-inclusion complexes or uncomplexed drugs, making CD-NSs a promising nanocarrier system [142]. In the light of progress on the above, it can be concluded that CDs, have worked miracles to shed cancer while safely avoiding chemotherapy.

### 3.6. Antioxidant Activity

Several compounds with antioxidant properties such as stilbenes, vitamins, carotenoids, coenzyme Q10, and fatty acids are complexed with CDs to increase their stability [143]. Curcumin, a natural antioxidant with poor water solubility, was encapsulated by β-CD. A strong improvement in curcumin’s solubility was observed. In vitro release of curcumin presented a faster release trend, after 10 h, when it is uncomplexed form reached 92.8% as compared to 63.67% in a complexed form. An explanation is the complexation of curcumin in the inner cavity of β-CD, which enables the protection of curcumin against different agents such as oxidation [144]. Further, one study developed the curcumin/β-CD polymer as a novel antioxidant with prospective utilization in cancer chemoprevention. In vitro anticancer activity results expressed stronger inhibitory effects of the curcumin/β-CD polymer on cancer cells, for 72 h, compared to free curcumin [145]. Antioxidant activity together with several others led to further studies for improving the stability, solubility, and pharmacokinetics of curcumin. A significant step forward for it came from CD-NSs. The curcumin was highly encapsulated in the CD-NS (82.81–94.38%), enhancing its solubility thanks to the reduction of particle size. Moreover, the release profile of curcumin was faster for curcumin-CD-NSs, increasing in this way the cytotoxicity effect on cancer cells [136].

Additionally, quercetin, best known for its antioxidant activity, was complexed with β-CD by performing electrospinning of polyacrylic acid (PAA) nanofibers (NF). The release profile of quercetin from the inclusion complexes of β-CD/PAA/NF/quercetin was 57% after 30 min and 97% after 48 h [68]. Further, quercetin-loaded CD-NSs were prepared using the freeze-drying technique. As usual, a faster dissolution of the drug was observed when it is encapsulated in CD-NSs. However, the crosslinking ratio influenced the release of quercetin from CD-NSs with 92–98% after 24 h. According to the type of CD-NSs, the release kinetics may be prolonged, with the molar ratio 1:4 or 1:6 (CD:linker) being the most appropriate for this drug [140].

Rutin [146], caffeic acid [147], vitamin E [148], astaxanthin [149], kynurenic acid [150], and resveratrol [151] were other compounds that present the CDs as a promising platform to affect the antioxidant activity, either by inclusion complexes or as polymeric drug delivery systems.

### 3.7. Diabetes Activity

In diabetic disease, for a better function of oral insulin administration, Song et al., in 2018, fabricated carboxymethyl-β-cyclodextrin-grafted chitosan nanoparticles (insulin-CMCD-g-CS NPs) via the ionic crosslinking method. The result of the study demonstrated that the oral administration of insulin-loaded CMC-g-CS nanoparticles declined the level of blood sugar in the mice model [152]. In addition, CD-NSs were proposed as insulin carriers too [153]. The complexes (CD-NSs/insulin) not only reduced the release at gastric pH of insulin but also enhanced the in vitro bioavailability, and the presence of insulin was in vivo confirmed. In a study, Ohira et al. compared two methods for the treatment of diabetic macular edema [154], where dexamethasone γ-cyclodextrin nanoparticle eye drops were prepared and considered. The results demonstrated that the nanoparticle was more effective in the improvement of visual acuity than triamcinolone acetonide and reduced macular thickness in patients with diabetic macular edema.

## 4. Discussions

The administration of poorly soluble drugs is a challenging step in any therapy. The use of CD monomers and polymers is a good tool to solve this issue as shown by the different examples presented in this review. However, if the carrier only “transports” the material, why did the complex sometimes present higher activity? Is it only a solubility effect? The complexation is an advantage to increase the stability and the establishment of a pure drug reservoir preventing its degradation by different physicochemical agents (pH, temperature, ROS). This effect can be further increased when CD-based polymers are synthesized. These polymers because of their complex 3D structure can efficiently protect the drug from degradation by slowing down its release. On the other hand, an increase in bioavailability is usually observed when the drug is complexed. Principally, the higher the drug solubility is, the more it enables the drug to easily cross barriers or be delivered to the target point. Therefore, all the above reasons can justify the higher activity presented by complexed drugs compared to that of free drugs at the same concentration.

On the other hand, CD monomers and polymers can present intrinsic bioactivity. The ability to complex different metabolites or to interact with different pathways may generate an “active” excipient in drug formulation. In this review, a particular case is an antiviral drug known as ganciclovir. Although the complex is formed with a 1:1 ratio, it displayed higher antiviral activity at a 1:10 ratio. This scope suggests that the activity of the carrier itself should be taken into account when the target is suitable to interact with it (as in the case of cholesterol of the membranes). As a consequence, a higher quantity of CD monomers and polymers in the formulation can generate an extra effect in treatment. To sum up, different points such as the increase of stability, bioavailability, or the intrinsic carrier activity are presented as a good explanation for the unexpected activity increase.

## 5. Conclusions

The present review emphasizes the role of CDs and CD-based polymers for enhancing bioactivities. Fundamentally interesting was not only the drug solubility but also the clarification of diverse activities such as antimicrobial, anticancer, antiviral, etc. In certain models, the photostability and bioavailability were improved achieving more effect on the target disease. On the other hand, several examples demonstrated an increase of complexed drug bioactivity (for example, antioxidant) in comparison to free drug, even though the concentration was kept uniform. In this survey, the action of CDs as active drugs was furthermore explored, which can suggest a combinatorial against various diseases. Moreover, as previously highlighted, the capacity to modulate the CD-based polymers is a good alternative to achieve a better release or target delivery than CD monomers.

Remarkably, this review indicates that not only the concentration but also different bioactivities can be improved if the inclusion complex is formed. In simple terms, the application of CD-based polymers is an empowering and significant progress in the last several years and has laid the groundwork for future progress opening up a new realm of other advanced applications expected to arise soon due to the high versatility of CD derivatives and novel synthetic types of CD polymers.

## Figures and Tables

**Table 1:** Examples of CD monomers or polymers activities by themselves.

Disease	Classic CD Monomer	CD Polymeric Material	Drug	Activity	Reference
Antimicrobial	Several CDs (HPβ- or α-CD)	-	trans-Anethone	- Improved solubility - Improved stability	[59]
	β-CD	-	Oxyresveratrol	- Increased solubility enhancing the antimicrobial capacity	[19]
	Several CDs (HPβ- or α-CD)		Neochlorogenic acid	- Improved solubility - Improved stability	[9]
	-	PS fibers containing CDs	Menthol	- Improved stability and durability	[61]
		Silver particles stabilized by β-CD	Silver particles	- Stabilizer of the particles	[60]
	-	β-CD polymers of polyethylene	Carvatrol	- Enhanced antimicrobial activity	[57]
	-	β-CD polymer with chitosan			[62]
	-	β-CD polymer with cellulose			[63]
	-	HPβ-CD electrospun nanofibers	Thiabendazole	- Improved solubility - Improved stability - Improved activity	[67]
			Thiram		[66]
	-	HPβ-, Mβ-, and HPγ-CD electrospun nanofibers	Limonene	- Improved solubility - Improved stability - Improved activity	[68]
	-	CD-NS	Coriander essential oil	- Improved release	[69]
	-		Babchi essential oil		[71]
	-		Cinnamon oil		[70]
	-		Norfloxacin	- Increased in vivo antibiotic capacity and permeability	[72]
	-		Lysozyme	- Absorption of the enzyme	[73]
	Several CDs (HPβ- or β-CD)	-	Citronella	- Improved release - In combination with Glucobay^®^, increased antimicrobial capacity	[58]
Antiviral	CD monomers (β-CD or HPβ-CD)	-	Acyclovir	- Increased solubility	[76]
		-	Efavirenz	- Increased solubility - Increased bioavailability	[75],
		-	Lopinavir		[77]
		-	Ganciclovir		[78]
	-	CD-NS	Efavirenz	- Increased bioavailability in rats	[79]
	-		Rilpivirine	- Increased bioavailability in rats	[80]
	-		Nelfinavir	- Increased solubility	[81]
	-		Acyclovir	- Increased antiviral activity against HSV-1	[82]
	-	Hyaluronic acid–CD covalent conjugates		- Novel way to deliver	[83]
		HPβ-CD electrospun nanofibers		- Improved release	[84]
	-	Sulfobutyl ether-β-CD decorated with nanodroplet chitosan		- Increased antiviral activity against HSV-2	[85]
Cardiovascular	β-CD	-	Linalool	- Decreased arterial pressure more than free drug (in β-pinene, free drug effect is negligible)	[87]
		-	β-Pinene		[88]
	β-CD in lipid nanoparticles	-	Hydrochlorothiazide	- Increased bioavailability	[90]
	-	HPβ-CD and SBEβ-CD in PVP polymers		- Increased solubility - Increased stability	[91]
Alzheimer’s disease	β-CD and HPβ-CD	-	β-Amyloid peptides	- Prevented aggregation	[97,98,99]
	-	Co-assembled CD/calixarene			[100]
	-	LK7-β-CD			[101]
	γ-CD	-	Crocetin	- Increased delivery	[102]
	HPβ-CD	Chitosan-coated polylactic polymer	Curcumin	- Increased stability	[103]
Parkinson’s disease	Mβ-CD	-	α-Synuclein	- Prevented aggregation	[105]
	-	CD-NS	L-Dopa	- Better controlled release - Improved stability	[107]
Diabetes	-	CD-NS	Insulin	- Increased stability - Improved release - Improved bioavailability	[153]
Anticancer	HPβ-CD	-	Niclosamide	- Improved solubility and bioavailability	[124]
		-	13-cis-Retinoic acid	- Improved bioavailability	[120]
	SBEβ-CD	-	Resveratrol	- Improved oral and parenteral bioavailability	[113]
		-	Celecoxib	- Improved the cytotoxicity of gemcitabine	[155]
		-	Erlotinib	- Increased apoptosis and inhibited autophagy	[156]
	-	CD-NS	Paclitaxel	- Enhanced water solubility and anticancer activity	[157]
	CD conjugates	-	Scutellarin	- High antiproliferative activities	[158]
	α-CDs	-	Oleanolic acid	- Induction of apoptosis of cancer cells	[159]
	-	HA/EDA/β-CD	Doxorubicin	- Localized chemotherapy of solid tumors.	[160]
	CD/PRs	-	10-Hydroxycamptothecin	- Effectively suppressed tumor growth	[161]
	-	CD-NS	Babchi oil	- Increased the solubility, photostability, and safety	[71]
	-	CNTs	Curcumin and doxorubicin hydrochloride	- Enhanced the therapeutic efficacy of drugs	[162]
	-	β-CD polymer	Sorafenib	- Increased the bioavailability and reduced the systemic toxicity	[131]
	β-CD	-	Vitexin	- Increased the bioavailability and dissolution	[163]
Antioxidant	HP-β-CD	-	Clove essential oil	- Increased the total phenolic content and antioxidant activity	[164]
		-	Hesperidin and hesperetin	- Increased the solubility	[165]
		-	Myricetin	- Increased solubility - Improved oral bioavailability and antioxidant activity	[166]
	β-CD	-	Tea catechins	- Affected the antioxidant reactivity	[167]
		-	Rosmarinic acid	- Enhanced the free radical scavenging ability and the storage stability	[168]
		-	Chrysin	- Increased the antioxidant potential	[169]
		-	Anthocyanins	- Improved bioavailability	[170]
	PLA/HP-β-CD/	-	Gallic acid	- High antioxidant activity	[171]
	-	CD-NS	Gamma-oryzanol	- Increased its potential as carrier	[172]
	-		Resveratrol	- Increased the oral bioavailability	[173]

## Data Availability

Not applicable.
